# Early and reversible changes to the hippocampal proteome in mice on a high-fat diet

**DOI:** 10.1186/s12986-019-0387-y

**Published:** 2019-08-23

**Authors:** Fiona H. McLean, Fiona M. Campbell, Domenico Sergi, Christine Grant, Amanda C. Morris, Elizabeth A. Hay, Alasdair MacKenzie, Claus D. Mayer, Rosamund F. Langston, Lynda M. Williams

**Affiliations:** 1 Division of Neuroscience, University of Dundee, Ninewells Hospital & Medical School, Dundee, DD1 9SY UK; 2 Rowett Institute, University of Aberdeen, Foresterhill, Aberdeen, AB25 2ZD UK; 3 Institute of Medical Sciences, University of Aberdeen, Foresterhill, Aberdeen, AB25 2ZD UK; 4 Biomathematics and Statistics Scotland, University of Aberdeen, Aberdeen, AB25 2ZD UK

**Keywords:** Hippocampus, Proteomics, Mice, High-fat diet, Dendritic morphology

## Abstract

**Background:**

The rise in global obesity makes it crucial to understand how diet drives obesity-related health conditions, such as premature cognitive decline and Alzheimer’s disease (AD). In AD hippocampal-dependent episodic memory is one of the first types of memory to be impaired. Previous studies have shown that in mice fed a high-fat diet (HFD) episodic memory is rapidly but reversibly impaired.

**Methods:**

In this study we use hippocampal proteomics to investigate the effects of HFD in the hippocampus. Mice were fed either a low-fat diet (LFD) or HFD containing either 10% or 60% (Kcal) from fat for 3 days, 1 week or 2 weeks. One group of mice were fed the HFD for 1 week and then returned to the LFD for a further week. Primary hippocampal cultures were challenged with palmitic acid (PA), the most common long-chain saturated FA in the Western diet, and with the anti-inflammatory, n-3 polyunsaturated FA, docosahexaenoic acid (DHA), or a combination of the two to ascertain effects of these fatty acids on dendritic structure.

**Results:**

HFD-induced changes occur in hippocampal proteins involved in metabolism, inflammation, cell stress, cell signalling, and the cytoskeleton after 3 days, 1 week and 2 weeks of HFD. Replacement of the HFD after 1 week by a low-fat diet (LFD) for a further week resulted in partial recovery of the hippocampal proteome. Microtubule-associated protein 2 (MAP2), one of the earliest proteins changed, was used to investigate the impact of fatty acids (FAs) on hippocampal neuronal morphology. PA challenge resulted in shorter and less arborised dendrites while DHA had no effect when applied alone but counteracted the effects of PA when FAs were used in combination. Dendritic morphology recovered when PA was removed from the cell culture media.

**Conclusion:**

This study provides evidence for the rapid and reversible effects of diet on the hippocampal proteome and the impact of PA and DHA on dendritic structure.

**Electronic supplementary material:**

The online version of this article (10.1186/s12986-019-0387-y) contains supplementary material, which is available to authorized users.

## Background

The underlying causes of spontaneous Alzheimer’s disease (AD) are not fully understood [1]. While a number of gene polymorphisms are associated with increased risk of AD, there is mounting evidence that environmental factors, particularly obesity, type 2 diabetes and an unhealthy diet, are major driving forces in its development [2, 3]. The current global obesity epidemic is mirrored by an increasing incidence of AD which is projected to quadruple by 2050 [4, 5]. This growing strength of association between diet and AD highlights the importance of understanding mechanisms linking nutrition and cognition.

Abnormal protein regulation in the hippocampus is a major pathological hallmark of AD, mainly in the form of β-amyloid plaques (Aβ) and neurofibrillary hyperphosphorylated tau tangles (NFT) [1, 6]. However, loss of dendritic arborisation [7] and synaptic failure are both reported to occur prior to the formation of Aβ and NFT [8] suggesting that earlier, as yet unidentified, events underlie the onset of this disease. Thus, it is important to identify these pathophysiological processes and determine what drives them.

Animal studies show that a high-fat diet (HFD) leads to obesity and causes cognitive impairment [9–11]. These studies and others have mostly looked at the relatively long-term impact of a HFD on memory from around 5 days onwards. However, we have recently shown rapid cognitive decline in hippocampal-dependent episodic memory, in mice, within 1 day of a HFD, which persisted for the time that animals remained on the diet [12]. Nonetheless, when mice were switched back to a low-fat diet (LFD) episodic memory was restored. In order to understand the potential mechanisms underlying the rapid and reversible onset of this cognitive impairment, changes in the hippocampal proteome were examined in a cohort of mice from the same experiment. Primary hippocampal cell culture was then used to confirm the influence of different dietary fatty acids (FAs) on dendritic morphology.

## Methods

### Animal study

Male, 12 week old, C57Bl/6 J mice (Harlan Laboratories, UK) were used for proteomic studies and Sprague-Dawley rat pups (*n* = 8) were used for primary hippocampal cultures. All studies adhered to UK Home Office regulations according to the Animals (Scientific Procedures) Act, 1986, were in accordance with the European Directive on the Protection of Animals used for Scientific Purposes 2010/63/E, and followed ARRIVE guidelines. Experimental protocols were approved by the Rowett Institute’s Ethical Review Committee. Animals were singly housed on grid floors and maintained on a 12:12 h light:dark cycle with access to food and water ad libitum. Environmental enrichment was provided.

Low-fat diet (10% energy from fat) D12450B and high-fat diet (60% energy from fat) D12492 (Research Diets Inc. New Jersey, US) were used. Mice were fed a LFD for 8 days to acclimatise to a semi-purified diet and then either remained on the LFD for a further 2 weeks or were switched to a HFD for 3 days, 1 week or 2 weeks or were fed a HFD for 1 week then switched back to a LFD for a week (high-fat diet recovery (HFDR)). Mice were counterbalanced between groups based on body weight at the beginning of the experiment (*n* = 6 mice per diet group per time point). Mice were euthanised by an intraperitoneal injection of 200 mg/ml pentobarbital sodium (Dunlops, UK) per kg body weight followed by decapitation and dissection. Brains were then snap-frozen on dry ice as previously described [12].

### Body weight and food intake measurements

Body weight and food intake were measured 3 times weekly and on the day of diet switch.

### Sample preparation

Whole mouse brains were stored at − 80 °C until dissection. Hippocampi were removed and tissue was weighed and homogenised in 40 mM Tris pH 7.4, 0.1% v/v Triton X-100 containing Roche complete protease inhibitors (Sigma Aldrich, UK) at a 1:4 tissue to buffer ratio. Protein concentrations of samples were calculated using a Pierce 660 nm Protein Assay (Thermo Fisher Scientific, UK). Samples were diluted with 15 μl DTT in a variable volume of buffer in order to load 200 μg of protein. Protein from a single mouse hippocampus was used for each gel.

### Two-dimensional gel electrophoresis (2-DE)

For separation of proteins in the first dimension, 17 cm immobilized pH gradient (IPG) strips (pH 3–10) (BioRad, US) were used. Strips containing 200 μg of protein sample were hydrated for 1 h at 20 °C in hydration buffer (7 M urea; 2 M thiourea; 4% w/v CHAPS; 2% w/v Biolyte; and 50 mM DTT) in an IEF cell (Bio-Rad, US) without applied voltage. Samples were isoelectrically focussed at 50 V/strip for 16 h and then incubated for 10–15 min at room temperature in equilibrium buffer (6 M urea; 2% w/v SDS; 0.375 M Tris-HCl, pH 8.8; 20% v/v glycerol; and 130 mM DTT) followed by incubation for 10–15 min at room temperature in a second equilibration buffer (6 M urea; 2% w/v SDS; 0.375 M Tris-HCl, pH 8.8; 20% v/v glycerol; and 135 mM iodoacetamide). Strips were loaded on to an 18x18cm gel cassette with 5 μl of All Blue Precision Protein Standards (Bio-Rad, US) as a reference and run at 200 V for 9.5 h or until the bromophenol blue had reached the bottom of the gel. Coomassie Blue (Brilliant Blue G, Sigma-Aldrich, UK) was then used to fix and stain the gels. After staining gels were transferred to a clean tray containing deionised H_2_O and any residual Coomassie Blue was removed. Gels were then shaken for 2 h in fresh deionised H_2_O which was replaced every hour. Gels were scanned and sealed in a plastic bag with approximately 5 ml of deionised H_2_O to prevent dehydration until spots were excised.

### Image analysis

Images of the 2-DE gels were analysed using PDQuest 2-D Analysis Software (Bio-Rad, US). Average optical densities of spots were measured and compared using Student’s t-test at each time point. Each group had 6 gels per group corresponding to one gel per animal except the HFD and corresponding LFD control group at 2 weeks which had 5 gels per group as one gel from each group was removed due to low protein loading. Spots which showed significant differences between groups (*p* < 0.05) were manually cut from gels using a modified pipette tip.

### Mass spectrometry

Isolated spots were trypsinized using the MassPrep Station (Waters, Micromass, UK) protocol. Tryptic peptides were then analysed by LC-MS/MS as previously described [13, 14] using ‘Ultimate’ nanoLC system (LC Packings, UK) and a Q-Trap (Applied Biosystems/MDS Sciex, UK) triple quadrupole mass spectrometer fitted with a nanospray ion source.

### Protein identification, functional classification and pathway analysis

Protein identifications were made by entering the total ion current (TIC) data into the MASCOT search engine (Matrix Science, UK) using the MSDB database. Functional classification and protein information was retrieved from the Uniprot database http://www.uniprot.org). Where proteins fall under more than 1 functional classification the protein is included in both. Uniprot accession numbers were then entered into Reactome (http://www.reactome.org) to establish the pathways and reactions associated with the proteins.

### Primary hippocampal cell cultures

Primary hippocampal cell cultures were derived from postnatal day 2 (P2) Sprague-Dawley rat pups (*n* = 8). The hippocampi were dissected, pooled and incubated in 2 ml trypsin for 15 min at 37 °C. Trypsin was replaced with 2 ml soybean trypsin inhibitor (Sigma-Aldrich, UK) for 5 min at room temperature. Soybean trypsin inhibitor was then replaced with 3 ml of supplemented media (Neurobasal-A medium with 2% B27 supplement, 1% Glutamax and 1% Penicillin/Streptomycin, all Gibco, UK). Tissue was triturated gently using a fire polished glass pipette to dissociate the tissue then media was removed and passed through a cell sieve into collection tube. Two ml of media was added to the remaining tissue then the process repeated approximately another 10 times. Cells were then plated onto poly-L-lysine coated coverslips in 24 well plates at a density of 180,000 cells per well. Cells were cultured on coverslips in medium (Neurobasal-A medium with 2% B27 supplement, 1% Glutamax and 1% Penicillin/Streptomycin, all Gibco, UK) at 37 °C and 5% CO_2_ for 7 days.

### Fatty acid conjugation

Both palmitic acid (PA) and docosahexaenoic acid (DHA) were conjugated to standard FA-free bovine serum albumin (BSA) (Sigma, Catalogue Number A6003) as described previously [15] with some alterations [16]. Fatty acids (FA) were dissolved in 0.1 M NaOH at either 37 °C or 70 °C for DHA or PA respectively in a shaking water bath to give a final concentration of 20 mM. FA-free BSA was dissolved at 55 °C in deionised water. The 20 mM FA solution was mixed with BSA to obtain a 1:4 BSA:FA molar ratio (2 mM FA: 0.5 mM BSA) as previously reported [17] and then vortexed for 10 s. The mixture was then incubated in a shaking water bath at 37 °C or 55 °C for DHA or PA, respectively. Mixtures were brought back to room temperature, filter sterilised using a 0.22 μM pore size membrane then stored at − 20 °C. FA conjugates were then diluted in culture medium to give the appropriate working concentrations.

### Fatty acid challenge

After 7 days in culture, primary hippocampal cell cultures were challenged with either 200 μM PA, 200 μM DHA, 200 μM PA plus 125 μM DHA or BSA as a control for 6 h. Cultures were also challenged with either BSA or PA for 3 h. To ascertain whether or not recovery from PA occurs, cells challenged with PA for 3 h were washed before incubation with BSA for a further 3 h. To ensure there was no effect of the washing procedure on experimental outcomes, cells incubated for 3 h with BSA or PA were washed and treatments were reintroduced for a further 3 h.

### Immunocytochemistry

After FA challenge, the plates were placed on ice, media was removed and the cells were fixed with ice-cold 4% paraformaldehyde for 20 min, washed in PBS (3 X 5 min) then permeabilised with 0.2% Triton X-100 for 15 min. Cells were washed 3 times with PBS. Non-specific staining was blocked with 8% BSA dissolved in PBS for 20 min. Cells were incubated in primary antibody rabbit anti-MAP2 (Abcam, UK) at [1:1000] for 1 h followed by 3 washes in PBS. Cells were then incubated in secondary antibody goat anti-rabbit Alexa Fluor 488 (Molecular Probes, USA) at [1:1000] for 1 h. Both primary and secondary antibodies were diluted in PBS containing 4% BSA and 0.2% Triton X-100. Control slides omitted the primary antibody which resulted in a complete lack of staining (data not shown). Coverslips were mounted onto glass slides using Vectashield (Vector Laboratories, UK). Cells were imaged using a Leica DMR microscope fitted with a QImaging QICAM FAST 1394 digital camera.

### MAP2 quantifications

Staining was analysed in images capture by fluorescence microscopy using WIS-NeuroMath software [18, 19]. This software allows for accurate measurements of cell morphology. Ten neurons per treatment were analysed.

### Statistics

One-way ANOVAs were carried out (using the Statistical Package for the Social Sciences (SPSS), USA) to compare neuronal area, number of dendrite branches from the cell body, average length of projections and number of branching points. When there were more than two groups, a Least Significant Difference (LSD) post-hoc comparison was carried out. Significance was *p* ≤ 0.05.

## Results

### Body weight and food intake

**Fig. 1:**
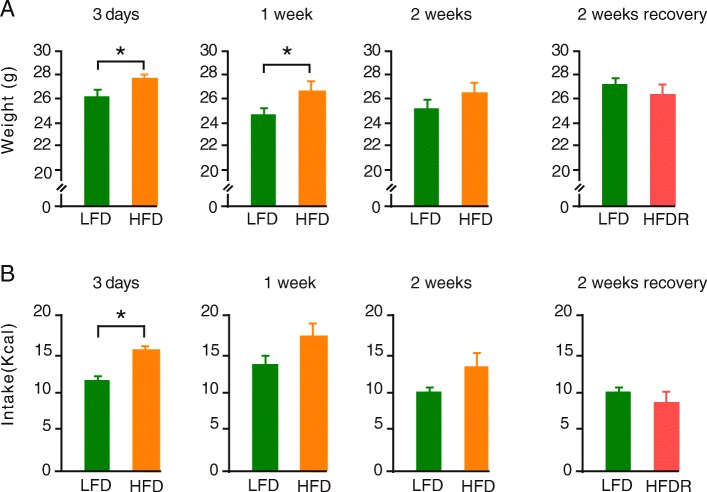
Body weight (g) of mice at **a**. 3 days**,** 1 week, 2 weeks and 2 weeks HFDR. **b**. Food intake (kcal) of mice at 3 days, 1 week, 2 weeks and 2 weeks HFDR. Data is presented as mean ± SEM (* *p* < 0.05,). Low-fat diet (LFD), high-fat diet (HFD) and high-fat diet recovery (HFDR)

Mice on a HFD showed increased body weight and were significantly heavier (*p* < 0.05) than the LFD mice at day 3 and 1 week of diet and had a significantly higher calorie intake at day 3 (p < 0.05). When HFD mice were switched to a LFD (high-fat diet recovery - HFDR), caloric intake was the same as those on a LFD (Fig. 1a-h).

### Hippocampal proteome on a high-fat diet (HFD) and HFD recovery (HFDR)

**Fig. 2:**
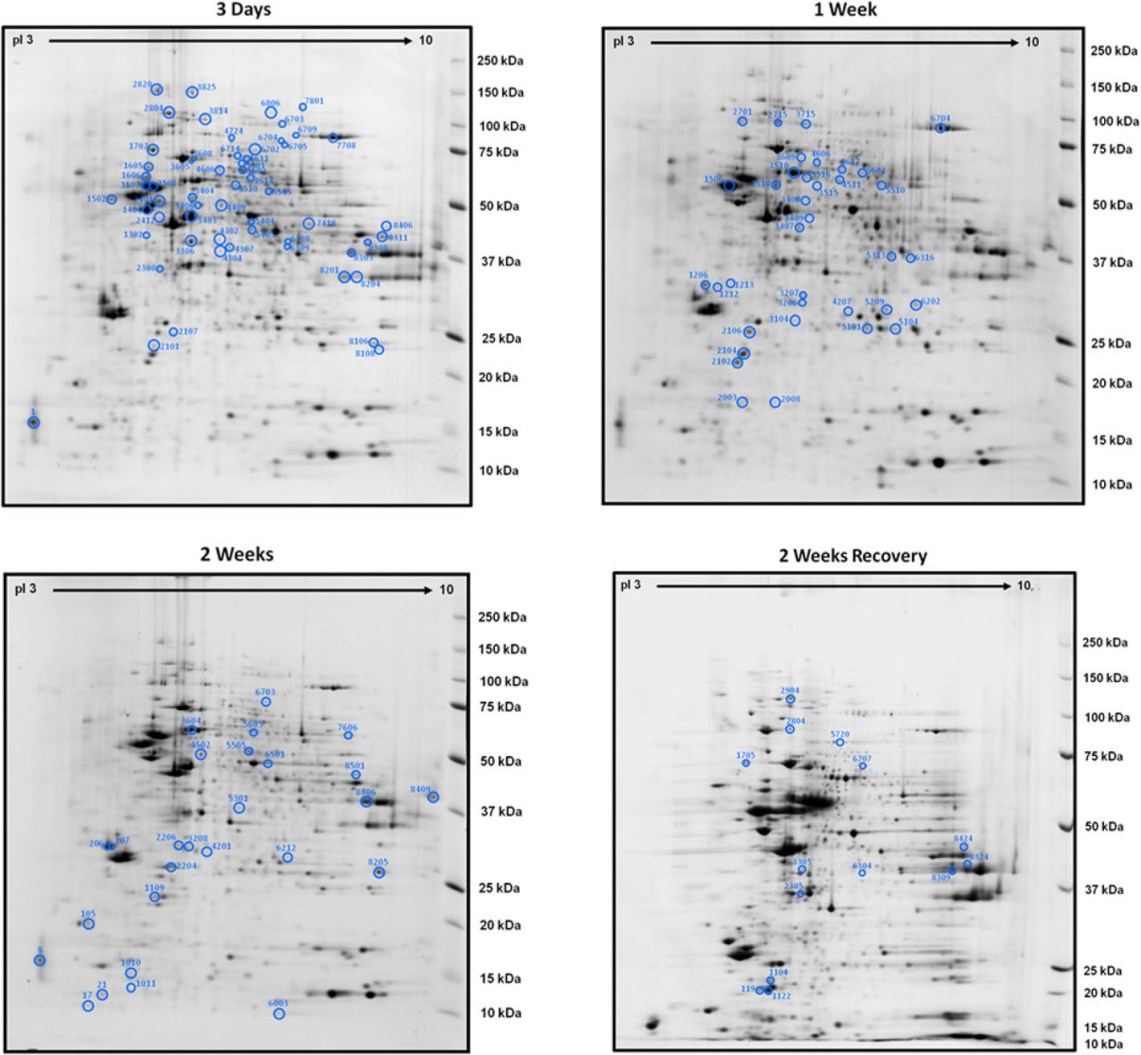
Images of two-dimensional gel electrophoresis (2-DE) of hippocampal proteins after a HFD with changed spots circled at 3 days, 1 week, 2 weeks and 2 weeks recovery. Numbers on gels refer to spots found in Additional file 1: Tables S1-S4

The HFD changed the pattern of hippocampal protein expression with 58 spots (46 unique proteins identified), 36 spots (28 unique proteins identified) and 27 spots (22 unique proteins identified) being altered compared to mice on a LFD after 3 days, 1 and 2 weeks respectively. When mice were switched after 1 week of HFD back to a LFD for 1 week (high-fat diet recovery - HFDR), 14 spots (11 unique proteins identified) were significantly altered compared to those on a LFD (Fig. 2a-d; Additional file 1: Tables S1–S4, Additional file 4: Tables S1-S4 References).

### Pathway analysis

**Fig. 3:**
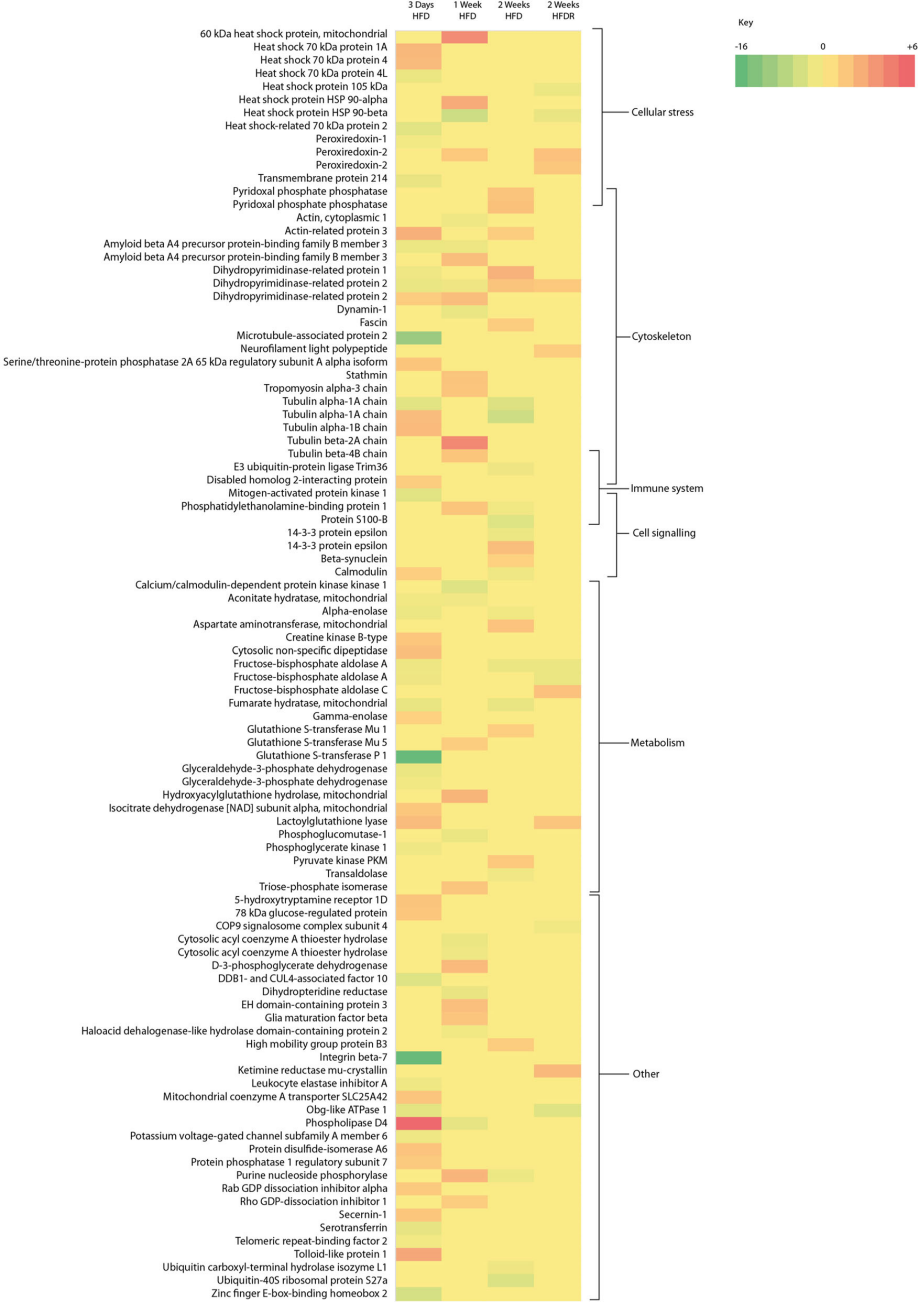
Heat map showing fold changes in proteins after 3 days, 1 week, 2 weeks of HFD and HFDR. Proteins are shown in multiples reflecting the number of spots which gave the same protein ID. High-fat diet (HFD) and high-fat diet recovery (HFDR)

**Fig. 4:**
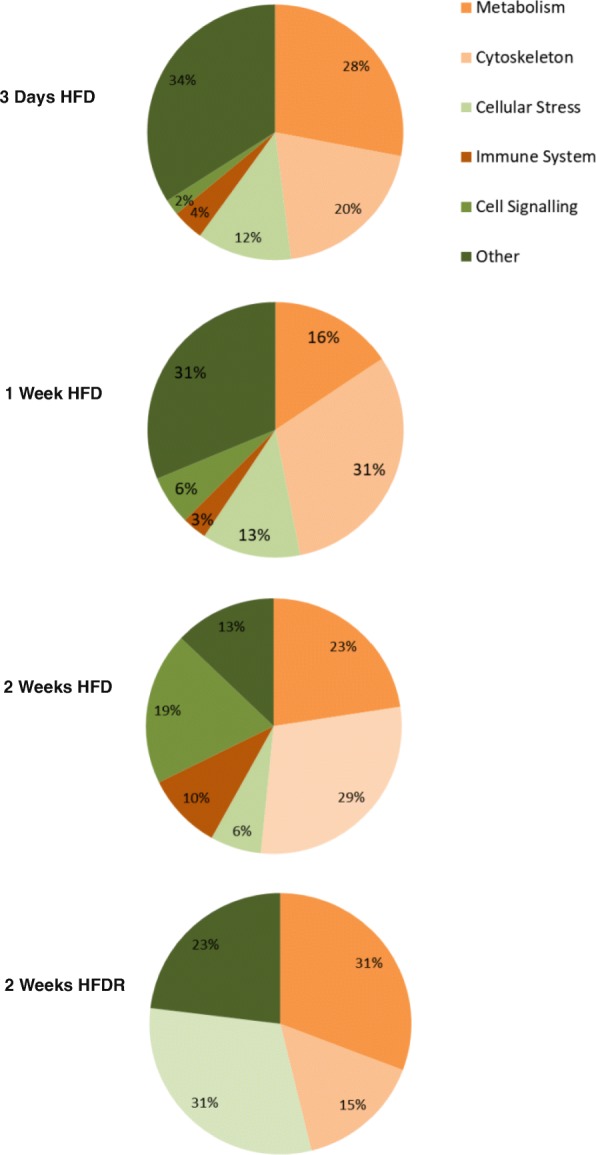
Pie charts showing the percentage of proteins changed from each functional group after 3 days, 1 week, 2 weeks HFD and HFDR. High-fat diet (HFD) and high-fat diet recovery (HFDR)

On a HFD, proteins related to metabolism, the cytoskeleton, cellular stress, the immune system and cell signalling were changed. On returning to a LFD after a HFD (HFDR) no proteins associated with the immune system or cell signalling remained changed (Figs. 3 and 4).

Reactome analysis identified the biochemical pathways with the most changed proteins. These included metabolism, signal transduction, axon guidance, developmental biology, carbohydrate metabolism, haemostasis, cell cycle, signalling by G Protein-Coupled Receptors (GPCR), the immune system and platelet activation, signalling and aggregation (Additional file 1: Tables S5–S8).

#### Neuronal area and number of proximal dendrites in primary hippocampal cell cultures

**Fig. 5:**
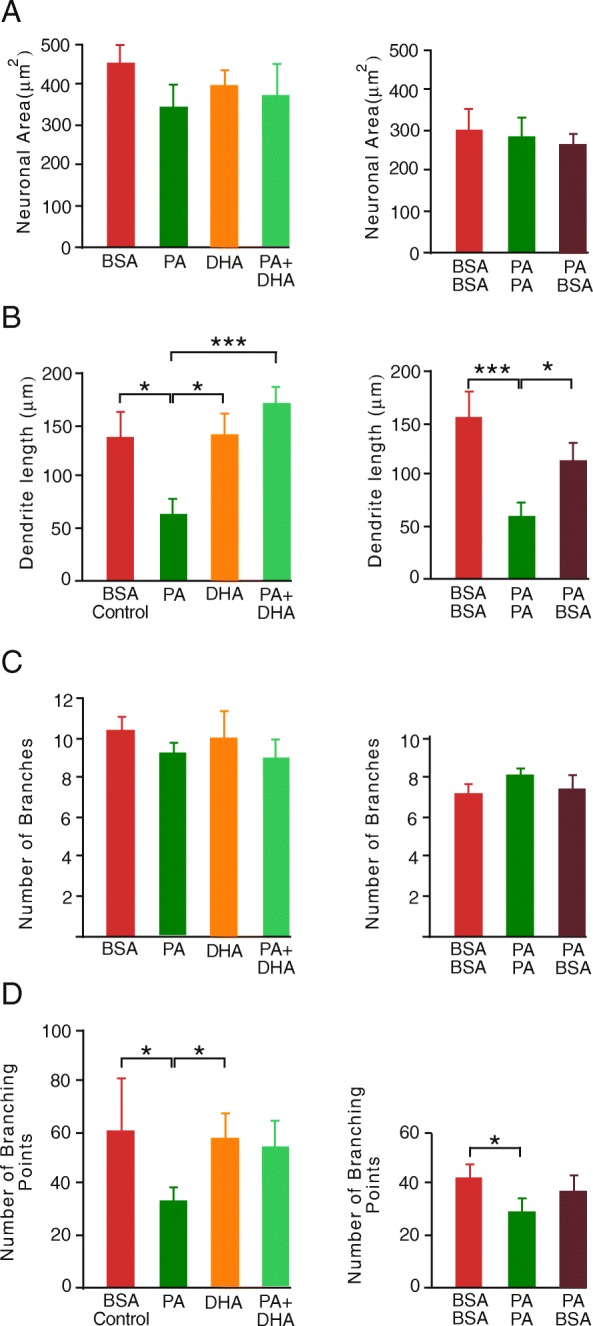
**a**. Area of microtubule-associated protein 2 (MAP2) immunostaining in primary hippocampal neurons in culture **b**. Dendrite length **c**. Mean number of dendritic branches originating from soma **d**. Number of dendritic branching points. Neurons were challenged with BSA (control), 200 μm PA, 200 μM DHA or 200 μM PA plus 125 μM DHA for a period of 3 h followed by a further 3 h challenge either with the same or different treatment (* *p* < 0.05, *** *p* < 0.001). Data presented as mean ± SEM, *n* = 10 all groups. Bovine serum albumin (BSA), palmitic acid (PA) and docosahexaenoic acid (DHA)

When primary hippocampal cell cultures were challenged with PA, DHA or a combination of the two FAs there were no differences in the area covered by MAP2 immunopositive neurons nor in the number of proximal dendrites from individual neurons compared to the control BSA group after 3 h (Additional file 2: Figure S1) and 6 h (Fig. 5a and c). There were also no differences in neuronal area in cells treated with PA followed by incubation with BSA (Fig. 5a and c).

#### Average length of dendrites in primary hippocampal cell cultures

The average length of dendrites was significantly shorter after both a 3 h (Additional file 1: Figure S1) and a 6 h challenge with PA compared to the BSA control (Fig. 5b). There was no difference in dendrite length in either DHA alone or in PA plus DHA challenged neurons relative to BSA-treated neurons. Additionally, dendritic length in neurons treated with PA followed by BSA was not different to BSA control (Fig. 5b). When investigating if cells could recover from a PA challenge, to ensure that any shearing forces resulting from changing solutions was ruled out as an influence on dendrite length, PA containing media and BSA containing control media were replaced after 3 h. The same differences between PA and BSA control were maintained under these conditions.

#### Number of dendrite branching points in primary hippocampal cell cultures

Hippocampal neurons challenged with PA had fewer branching points compared with BSA controls after both 3 h (Additional file 1: Figure S1) and 6 h challenge (Fig. 5d). There was no difference in the number of branching points between either DHA alone or PA plus DHA compared to BSA control. The number of branching points in neurons that were treated with PA followed by incubation with BSA was not different to BSA control (Fig. 5d). As described above changing solutions was ruled out as an influence on number of branching points as the same differences between PA and BSA control were maintained under these condition.

#### Dendritic morphology in primary hippocampal cell cultures

**Fig. 6:**
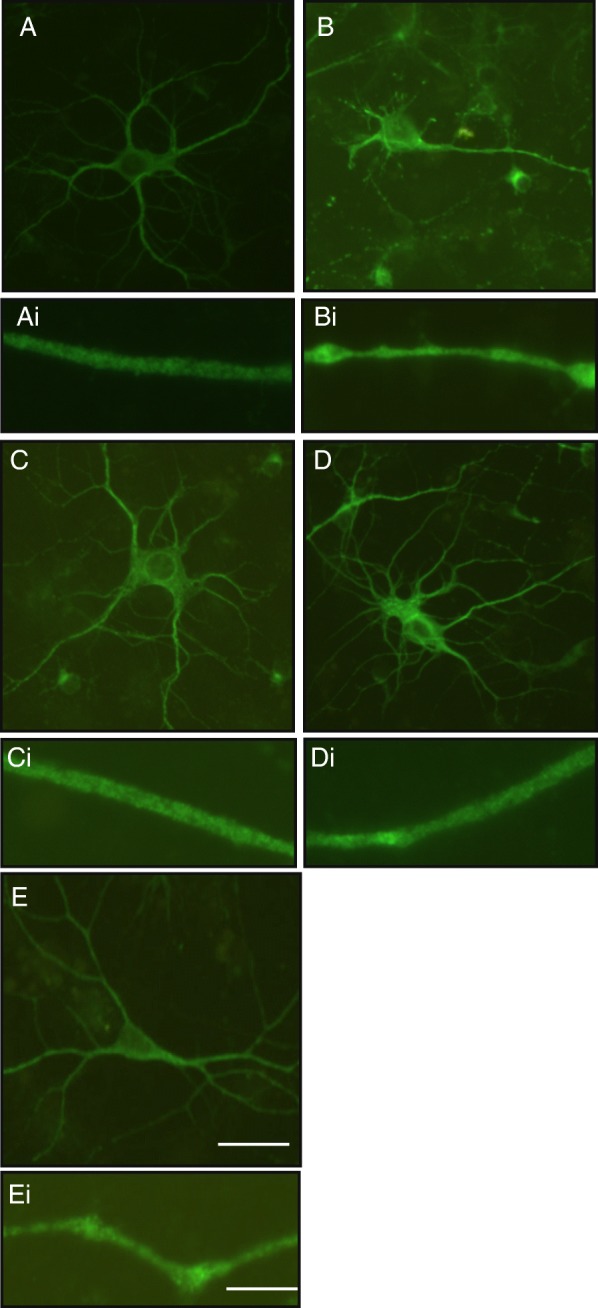
Representative images of microtubule-associated protein 2 (MAP2) immunostaining captured with fluorescence microscopy showing dendritic morphology in hippocampal neurons challenged for 6 h with: **A.** BSA alone (control) **B.** 200 μM PA **C.** 200 μM DHA **D.** 200 μM PA plus 125 μM DHA **E.** 200 μM PA for 3 h followed by BSA for 3 h. Scale bar: 20 μm. **Ai-Ei.** Higher magnification fluorescence microscopy of MAP2 immunostaining showing dendritic morphology in hippocampal neurons challenged for 6 h with: **Ai.** BSA alone (control) **Bi.** 200 μM PA **Ci.** 200 μM DHA **Di.** 200 μM PA plus 125 μM DHA. **Ei.** 200 μM PA for 3 h followed by BSA for 3 h. Bovine serum albumin (BSA), palmitic acid (PA) and docosahexaenoic acid (DHA). Scale bar: 10 μm. Bovine serum albumin (BSA), palmitic acid (PA) and docosahexaenoic acid (DHA)

Representative fluorescent images of MAP2 immunostaining showed dendritic morphology in hippocampal neurons challenged with both DHA alone and PA plus DHA had comparable morphology to BSA control neurons whilst neurons treated with PA showed reduced dendritic arborisation (Fig. 6a-e). Neurons treated with PA also showed dendritic blebbing consisting of localised swellings that gave a beads on a string appearance. Neurons challenged with PA plus DHA and DHA alone were comparable to BSA control with no dendritic blebbing (Fig. 6Ai-Ei). Neuronal challenges in which the PA was replaced by BSA still exhibited blebbing but to a lesser degree than when PA was present in the media (Fig. 6E and Ei).

## Discussion

Proteomic analysis demonstrates rapid changes in specific proteins and pathways in the hippocampus in response to a HFD, however, in the present study it is not possible to say whether these are due to changes in protein expression and/or turnover or post-translational modifications. Nonetheless proteins found to change the most in the present study cluster into diverse pathways involved in metabolism, cellular stress responses, cytoskeletal organisation, cell signalling and the immune system with almost half of the altered proteins also linked to aging, memory processes and neurodegeneration.

The fact that many of the proteins changed by a HFD are involved in metabolism is unsurprising due to the switch from carbohydrate to fats as the primary energy substrate. Thus, metabolic changes in the brain induced by a HFD underlie alterations in hippocampal metabolic enzymes implying an adaptation and perhaps impairment of cellular metabolism with lipid oversupply promoting neuronal inflammation and lipotoxicity [20]. Disruption of brain nutrient and energy metabolism has been established as a cause of cognitive impairment and central in the development of AD [21]. Hippocampal neurons are particularly susceptible to metabolic dysregulation as they often have long neurites which require more energy. This large surface area makes these neurons more prone to cytoskeletal impairment as well as increasing their risk of exposure to surrounding environmental conditions [22]. Therefore, neurons are susceptible to an increase in long-chain saturated fatty acid supply which may compromise efficient energy metabolism given the detrimental effect this can have on mitochondria, the power house of the cells [23].

One of the metabolic enzymes changed in response to a HFD is the glycolytic enzyme triose-phosphate isomerise, which has been linked to both Aβ and tau protein aggregation, with higher levels in AD patients [24, 25]. Others include, lactoylglutathione lyase and mitochondrial hydroxyacylglutathione hydrolase, two enzymes changed by a HFD which are involved in the detoxification of methylglyoxal which may be exacerbated in our model given the presence of both long-chain saturated fatty acids and refined carbohydrates in our experimental diet. Methylglyoxal is one of the most reactive glycation agents produced in vivo [26] glycating proteins, nucleotides and lipids and leading to the formation of advanced glycation end products (AGEs), which are causative in tissue damage seen in diabetes [27] and associated with AD [28]. Hippocampal neurons are particularly susceptible to methylglyoxal-induced neurotoxicity characterised by inflammation and apoptotic cell death [29, 30].

Another group of metabolic enzymes that show changes across all three time points on a HFD are the glutathione S-transferases. These proteins catalyse glutathione conjugation to a range of electrophilic compounds and have a role in cellular detoxification [31] by suppressing cellular levels of lipid peroxidation which can induce apoptosis [32]. Reduced activities of glutathione S-transferases in the hippocampus and related brain regions, as well as depleted protein levels in cerebrospinal fluid (CSF), have been found in AD patients [33]. The enzyme creatine kinase B-type maintains local ATP/ADP ratio by catalysing the transfer of high energy phosphate groups from ATP to phosphocreatine thus playing a pivotal role in brain cellular energy homeostasis. Not surprisingly, given the impact of a HFD on energy metabolism, this enzyme, was also changed by a HFD and is reported to be dysregulated and oxidised in AD [34, 35].

Many proteins identified as changed by a HFD are linked to cell stress and cell death, including a number of heat shock proteins (HSPs) which are changed by a HFD at all time points tested. These proteins respond to oxidative, mitochondrial and endoplasmic reticulum stress, all of which are known to be induced by a HFD [36]. HSPs act as molecular chaperons and have roles in correcting protein misfolding, improper protein aggregation, mediation of protein targeting to organelles and have a protective role in cellular regulation [37]. Changes in HSPs are indicative of cellular stress and can be interpreted as an attempt to reinstate protein homeostasis to prevent protein dysfunction.

Other enzymes involved in limiting neuronal damage include peroxiredoxin-1 (PRDX1) and peroxiredoxin-2 (PRDX2), both changed by a HFD. PRDXs are antioxidant, peroxide-scavenging proteins and as such have neuroprotective functions in inflammation and oxidative stress [38, 39]. Changes in both HSPs and peroxiredoxins in response to a HFD confirm HFD-induced protein misfolding, oxidative stress and neuronal cell death, key features of neurodegenerative diseases [38, 40].

Unexpectedly, a HFD also caused changes in hippocampal proteins involved in the cytoskeleton, particularly proteins which have roles in tubulin and microtubule (MT) stability including microtubule-associated protein 2 (MAP2) and stathmin. MT stability plays a crucial role in the organisation of neurites, cell communication and memory [41]. Proteins which regulate actin are also changed by a HFD including fascin and actin-related protein 3, which are important for the organisation of actin filaments. Actin filaments and actin polymerization are key in dendritic spine formation which are crucial for memory formation [42].

Both dihydropyrimidinase-related protein 1 (DRP-1) and dihydropyrimidinase-related protein 2 (DRP-2) were changed by a HFD. DRP-1 is key in dendritic spine morphology [43] with DRP-1 knock-out mice exhibiting reduced long-term potentiation in the hippocampus, deficits in hippocampal-dependent spatial memory tasks and decreased and disorganised MAP2 staining in the dendrites of hippocampal cells [44]. DRP-2 is also crucial for neutrite organisation [45, 46] and mouse studies have implicated hyperphosphorylated DRP2, driven by the overexpression of amyloid precursor protein (APP), as an early indicator of AD [47].

There were also HFD-induced changes in amyloid beta A4 precursor protein-binding family B member 3 (FE65L2), which binds to and processes APP [46, 48]. APP has been implicated in the regulation of neuronal actin-based membrane motility processes [49]. Taken together, these numerous changes in cytoskeletal organisation almost certainly influence neurite functionality changing neuronal communication required for learning and memory.

Neuroinflammation has previously been associated with a HFD and cognitive impairment [11, 50], this is supported by the present study which shows changes in proteins associated with inflammation. These proteins included S100-B, phosphatidylethanolamine-binding protein 1, E3 ubiquitin-protein ligase Trim36, disabled homolog 2-interacting (DAB2IP) and mitogen-activated protein kinase 1 (MAPK1). These findings add to the evidence implicating inflammation as a cause of cognitive dysfunction as demonstrated by the manifestation of long-term memory deficits in mice overexpressing inflammatory cytokines in the hippocampus [51] and provide further support to the putative role of neuroinflammation as a mediator of HFD-induced cognitive impairment.

Hippocampal proteins in mice recovering from a HFD (HFDR) revealed a proteome that had almost returned to control levels. However, it should be noted that the bodyweight and intake of mice at this time had already recovered to control levels, indicating that some alterations in the proteome involved in this recovery may have been missed. Of the 11 identified proteins which changed in HFDR, 5 have also been reported to change as a result of exercise including fructose-bisphosphate aldolase A (ALDOA), fructose bisphosphate aldolase C (ALDOC), peroxiredoxin-2, DRP-2 and neurofilament light polypeptide (NFL) [52]. Changes to the hippocampal proteome induced by switching from a HFD to a LFD and increasing exercise show some overlap, thus, demonstrating that exercise induced changes in hippocampal metabolism appear in part to resemble the metabolic switch from HFD to LFD.

To further investigate the impact of a HFD on the cytoskeleton, hippocampal primary cell cultures were challenged with different FAs and immunostained for MAP2. PA challenge reduced the length of dendrites, the number of dendritic branches and induced dendritic ‘blebbing’ consisting of localised swellings along the length of a dendrite which give the appearance of beads on a string. Blebbing is thought to be the result of the accumulation of proteins and membranous organelles due to cytoskeletal degradation usually seen in studies on excitoxicity and ischemia and is recognised as an early hallmark of neural toxicity [53, 54]. PA effects are rapidly reversed when PA is removed from the media. This supports our proteomic data where replacing a HFD with a LFD results in the return of the proteome to almost control levels.

Unlike PA, challenge with DHA did not change dendritic morphology and when administered at the same time as PA, DHA protected against the effects of PA. Several mechanisms may underlie this effect. Cell studies have shown that DHA is anti-inflammatory by decreasing the release of pro-inflammatory cytokines such as TNF-α, IL-6 and IL-8 and increasing release of the anti-inflammatory cytokine IL-10 [55]. Also, DHA exhibits antioxidant effects in rodent models of aging and AD [56]. Furthermore, n-3 polyunsaturated fatty acids have been shown to modulate fatty acid metabolism by increasing their catabolism via a mechanism which involves increased fatty acid β-oxidation within mitochondria [57]. Increased fatty acid catabolism may, in turn, counteract the detrimental effect exerted by PA on hippocampal neurons by preventing PA-induced neuronal toxicity and the build-up of lipotoxic lipid species such as ceramide [58]. Thus, the present study not only supports the fact that DHA is beneficial to neuronal function but demonstrates that it can counter the effects of PA.

## Conclusions

In conclusion, this study shows rapid and reversible changes in the hippocampal proteome in response to a HFD with approximately 40% of the proteins changed by a HFD also associated with AD [33, 35, 57–66]. These similarities provide further support for changes in energy metabolism as underlying the development of cognitive disorders with a HFD playing a key role in accelerating this process. A large number of the proteins changed by a HFD have cytoskeletal functions and primary hippocampal cell cultures challenged with PA show inhibition of dendritic arborisation and dendritic blebbing indicating changes in the cytoskeleton whereas DHA and the absence of PA can reverse these changes. Previous studies have implicated HFD in changes in the cytoskeleton [13, 67] and our study further bolsters this evidence by providing a mechanism through which a HFD can impact synaptogenesis, cognition and memory (Additional file 3: Figure S2). Further investigation is needed however, to elucidate processes involved in the interaction of a HFD with the cytoskeleton.

## Additional files

Additional file 1: **Tables S1–4.** Hippocampal proteins changed after 3 days on a HFD. **S2.** after 1 week on a HFD, **S3.** after 2 weeks on a HFD. **S4.** after 1 week of high-fat diet followed by 1 week of low-fat diet HFDR. **S5–8.** Top Reactome pathways mapped for hippocampal proteins changed after **S5.** 3 days on a HFD. **S6.** after 1 week on a HFD. **S7.** after 2 weeks on a HFD.**S8.** after 1 week of high-fat diet followed by 1 week of low-fat diet HFDR. High-fat diet (HFD), High-fat diet recovery (HFDR). (DOCX 45 kb)

Additional file 2: **Figure S1**. **A.** Area of MAP2 immunostaining in primary hippocampal neurons in culture **B.** Dendrite length **C.** Mean number of dendritic branches originating from soma **D.** Number of dendritic branching points. Neurons were challenged with BSA (control) or 200 μm palmitic acid PA solutions were changed and cells washed followed by a subsequent 3 h treatment with the same challenge (* *p* < 0.05, *** *p* < 0.001). Data presented as mean ± SEM, *n* = 10 all groups. Bovine serum albumin (BSA), palmitic acid (PA) and docosahexaenoic acid (DHA), microtubule-associated protein 2 (MAP2). (PDF 9 kb)

Additional file 3: **Figure S2.** A diagrammatic representation of the experimental design and major outcomes of the study. (TIF 624 kb)

Additional file 4: References for Tables S1-S4. (DOCX 23 kb)

## Data Availability

All data generated or analysed during this study are included in this published article [and its supplementary information files]. Additional data is available on request to the corresponding author.

## Abbreviations

AD: Alzheimer’s disease
AGEs: Advanced glycation end products
ALDOA: Fructose-bisphosphate aldolase A
ALDOC: Fructose bisphosphate aldolase C
APP: Amyloid precursor protein
Aβ: β amyloid plaques
BSA: Bovine serum albumin
CRMPs: Collapsin response-mediator proteins
CSF: Cerebrospinal fluid
DAB2IP: Disabled homolog 2-interacting protein
DHA: Docosahexaenoic acid
DRP1: Dihydropyrimidinase-related protein 1
DRP2: Dihydropyrimidinase-related protein 2
GPCR: G Protein-Coupled Receptors
HFD: High-fat diet
HFDR: High-fat diet recovery
HSPs: Heat shock proteins
LFD: Low-fat diet
MAP2: Microtubule-associated protein 2
MAPK1: Mitogen-activated protein kinase 1
NFL: Neurofilament light polypeptide
NFT: Neurofibrillary hyperphosphorylated tau tangles
PA: Palmitic acid
PRDX1: Peroxiredoxin-1
PRDX2: Peroxiredoxin-2
TIC: Total ion current
