# Supplementary material for: Early and reversible changes to the hippocampal proteome in mice on a high-fat diet
Source: Nutr Metab (Lond). 2019 Aug 23;16:57. doi: 10.1186/s12986-019-0387-y (PMC6708244; doi:10.1186/s12986-019-0387-y)
Supplement: Supplementary file 1 — Tables S1–4. Hippocampal proteins changed after 3 days on a HFD. S2. after 1 week on a HFD, S3. after 2 weeks on a HFD. S4. after 1 week of high-fat diet followed by 1 week of low-fat diet HFDR. S5–8. Top Reactome pathways mapped for hippocampal proteins changed after S5. 3 days on a HFD. S6. after 1 week on a HFD. S7. after 2 weeks on a HFD.S8. after 1 week of high-fat diet followed by 1 week of low-fat diet HFDR. High-fat diet (HFD), High-fat diet recovery (HFDR). (DOCX 45 kb) [file 12986_2019_387_MOESM1_ESM.docx]

**Table S1**

| **PD Quest spot number** | **UNIPROT accession number** | **Protein identification** | **p-value** | **Fold change** | **Average normalised quantity** | | **MASCOT score** | **Peptides matched** | **Sequence coverage (%)** | **pI, Mr (kDa)** |
| --- | --- | --- | --- | --- | --- | --- | --- | --- | --- | --- |
|  |  |  |  |  | **LFD** | **HFD** |  |  |  |  |
| 6505 | Q61224 | *^ref 1^ 5-hydroxytryptamine receptor 1D | 0.015 | 1.7 | 44.63 | 77.82 | 42 | 2 (1) | 3 | 6.48, 53890 |
| 1707 | P20029 | *^ref 2^78 kDa glucose-regulated protein | 0.017 | 1.6 | 79.96 | 123.84 | 133 | 9 (5) | 16 | 5.03, 79620 |
| 7708 | Q99KI0 | *^ref 3^ Aconitate hydratase, mitochondrial | 0.047 | 1.4 | 125.50 | 93.05 | 45 | 9 (2) | 14 | 7.67, 89960 |
| 3406 | Q99JY9 | *^ref 4^ Actin-related protein 3 | 0.030 | 2.6 | 20.60 | 54.44 | 122 | 7 (6) | 18 | 5.46, 48400 |
| 5404 | P17182 | *^refs 5^Alpha-enolase | 0.045 | 1.6 | 19.83 | 12.19 | 60 | 6 (3) | 16 | 6.14, 44840 |
| 6702 | Q8R1C9 | *^ref 6^Amyloid beta A4 precursor protein-binding family B member 3 | 0.023 | 1.7 | 29.50 | 17.23 | 40 | 3 (2) | 2 | 6.71, 82680 |
| 1 | P62204 | Calmodulin | 0.022 | 1.3 | 466.53 | 592.08 | 73 | 10 (2) | 32 | 4.09, 16830 |
| 3403 | Q04447 | *^refs 7,8^ Creatine kinase B-type | 0.019 | 1.5 | 1149.32 | 1765.99 | 464 | 59 (33) | 44 | 5.40, 45100 |
| 3404 | Q9D1A2 | Cytosolic non-specific dipeptidase | 0.005 | 2.0 | 36.92 | 72.82 | 146 | 11 (6) | 24 | 5.40, 51130 |
| 6709 | A2AKB9 | DDB1- and CUL4-associated factor 10 | 0.032 | 3.4 | 52.58 | 15.35 | 43 | 3 (2) | 4 | 7.14, 90790 |
| 4606 | P97427 | *^ref 9^ Dihydropyrimidinase-related protein 1 | 0.013 | 1.6 | 14.91 | 9.56 | 42 | 3 (1) | 3 | 5.69, 61210 |
| 4614 | O08553 | *^refs 10, 11^Dihydropyrimidinase-related protein 2 | 0.003 | 2.0 | 11.39 | 5.57 | 193 | 17 (9) | 29 | 5.86, 61650 |
| 5602 | O08553 |  | 0.040 | 1.4 | 291.56 | 403.54 | 1871 | 125(85) | 60 | 5.95, 62640 |
| 5601 | Q3UHC7 | Disabled homolog 2-interacting protein | 0.033 | 1.3 | 5.56 | 7.47 | 42 | 8 (2) | 3 | 5.94, 72410 |
| 8308 | P05064 | Fructose-bisphosphate aldolase A | 0.032 | 1.8 | 147.72 | 84.01 | 84 | 5 (2) | 20 | 8.52, 40810 |
| 8303 | P05064 |  | 0.048 | 1.5 | 216.56 | 145.60 | 196 | 15 (6) | 42 | 8.36, 39810 |
| 7418 | P97807 | Fumarate hydratase, mitochondrial | 0.009 | 2.1 | 92.68 | 43.23 | 47 | 3 (1) | 7 | 7.43, 44140 |
| 1404 | P17183 | Gamma-enolase | 0.045 | 1.2 | 1120.00 | 1306.06 | 515 | 57 (26) | 65 | 4.99, 47610 |
| 8106 | P19157 | *^ref 12^Glutathione S-transferase P 1 | 0.043 | 15.7 | 277.98 | 17.74 | 103 | 8 (3) | 31 | 8.67, 23710 |
| 8204 | P16858 | Glyceraldehyde-3-phosphate dehydrogenase | 0.029 | 1.8 | 234.70 | 128.28 | 152 | 12 (6) | 30 | 8.44, 36070 |
| 8201 | P16858 |  | 0.041 | 1.4 | 141.58 | 102.45 | 111 | 12 (7) | 20 | 8.15, 36060 |
| 3608 | Q61696 | *^refs 13, 14^Heat shock 70 kDa protein 1A | 0.043 | 2.2 | 116.16 | 260.56 | 71 | 3 (2) | 5 | 5.42, 70100 |
| 2804 | Q3U2G2 | *^refs 13, 14^Heat shock 70 kDa protein 4 | 0.019 | 2.1 | 31.31 | 66.50 | 108 | 8 (4) | 12 | 5.15, 127010 |
| 3814 | P48722 | *^refs 13, 14^Heat shock 70 kDa protein 4L | 0.015 | 2.0 | 9.42 | 4.72 | 44 | 3 (1) | 4 | 5.55, 114430 |
| 3605 | P17156 | *^refs 13, 14^Heat shock-related 70 kDa protein 2 | 0.031 | 3.0 | 41.13 | 13.86 | 255 | 9 (7) | 20 | 5.38, 69450 |
| 7801 | P26011 | Integrin beta-7 | 0.043 | 15.7 | 277.98 | 17.74 | 52 | 1 (1) | 2 | 7.20, 130140 |
| 3306 | Q9D6R2 | *^refs 15, 16^Isocitrate dehydrogenase [NAD] subunit alpha, mitochondrial | 0.025 | 1.6 | 87.66 | 140.71 | 180 | 14 (7) | 29 | 5.40, 40100 |
| 2101 | Q9CPU0 | *^ref17^Lactoylglutathione lyase | 0.038 | 2.1 | 34.20 | 71.18 | 56 | 4 (2) | 22 | 5.05, 23010 |
| 4302 | Q9D154 | Leukocyte elastase inhibitor A | 0.014 | 1.5 | 11.18 | 7.35 | 54 | 6 (1) | 15 | 5.71, 40450 |
| 6806 | P20357 | Microtubule-associated protein 2 | 0.027 | 10.0 | 40.15 | 4.03 | 38 | 4 (1) | 0 | 6.82, 114880 |
| 5405 | Q8R0Y8 | Mitochondrial coenzyme A transporter SLC25A42 | 0.013 | 1.6 | 24.39 | 39.73 | 42 | 1 (1) | 5 | 6.25, 43680 |
| 6308 | P63085 | Mitogen-activated protein kinase 1 | 0.003 | 3.0 | 68.98 | 22.96 | 51 | 2 (1) | 6 | 6.92, 40500 |
| 8406 | Q9CZ30 | Obg-like ATPase 1 | 0.030 | 2.6 | 149.29 | 58.50 | 40 | 5 (1) | 13 | 8.80, 43860 |
| 8108 | P35700 | *^ref 9^ Peroxiredoxin-1 | 0.016 | 1.2 | 212.69 | 181.92 | 39 | 6 (2) | 26 | 8.77, 22900 |
| 8311 | P09411 | Phosphoglycerate kinase 1 | 0.026 | 1.6 | 315.04 | 197.04 | 91 | 12 (5) | 34 | 8.72, 42290 |
| 2412 | Q8BG07 | Phospholipase D4 | 0.012 | 5.8 | 44.32 | 258.42 | 43 | 3 (1) | 7 | 5.11, 45010 |
| 4510 | Q61923 | Potassium voltage-gated channel subfamily A member 6 | 0.027 | 1.6 | 12.16 | 7.74 | 57 | 3 (2) | 2 | 5.75, 57390 |
| 2401 | Q922R8 | Protein disulfide-isomerase A6 | 0.003 | 1.8 | 151.87 | 268.04 | 116 | 7 (4) | 18 | 5.08, 50790 |
| 1302 | Q3UM45 | Protein phosphatase 1 regulatory subunit 7 | 0.045 | 1.4 | 43.33 | 62.59 | 55 | 1 (1) | 3 | 4.96, 42150 |
| 1606 | P50396 | Rab GDP dissociation inhibitor alpha | 0.016 | 1.5 | 316.67 | 467.57 | 181 | 20 (11) | 44 | 4.99, 59870 |
| 1502 | Q9CZC8 | Secernin-1 | 0.040 | 1.6 | 129.59 | 201.75 | 131 | 10 (6) | 21 | 4.78, 51410 |
| 1605 | Q76MZ3 | Serine/threonine-protein phosphatase 2A 65 kDa regulatory subunit A alpha isoform | 0.047 | 1.6 | 116.34 | 190.22 | 233 | 19 (11) | 30 | 4.98, 63540 |
| 6705 | Q921I1 | Serotransferrin | 0.006 | 2.4 | 26.29 | 10.77 | 58 | 4 (1) | 5 | 6.83, 83020 |
| 5611 | O35144 | Telomeric repeat-binding factor 2 | 0.025 | 1.2 | 7.47 | 6.22 | 44 | 2 (1) | 4 | 6.27, 73810 |
| 2820 | Q62381 | Tolloid-like protein 1 | 0.024 | 2.9 | 9.36 | 27.50 | 39 | 3 (1) | 2 | 5.13, 173900 |
| 6714 | Q8BM55 | Transmembrane protein 214 | 0.038 | 2.1 | 33.62 | 16.03 | 42 | 4 (2) | 1 | 6.55, 76170 |
| 4409 | P68369 | Tubulin alpha-1A chain | 0.023 | 2.9 | 7.51 | 2.59 | 41 | 1 (1) | 1 | 5.74, 50050 |
| 2501 | P68369 |  | 0.012 | 2.1 | 795.79 | 1638.63 | 445 | 40 (22) | 51 | 5.08, 55710 |
| 1507 | P05213 | Tubulin alpha-1B chain | 0.016 | 2.2 | 275.90 | 606.03 | 311 | 22 (14) | 44 | 5.00, 56130 |
| 6703 | Q9R0G7 | Zinc finger E-box-binding homeobox 2 | 0.045 | 4.2 | 70.03 | 16.57 | 40 | 7 (1) | 0 | 6.73, 105580 |
| 6704 | Non-Identifiable |  | 0.011 | 2.6 | 25.64 | 9.73 |  |  |  | 6.81, 86650 |
| 4304 | Non-Identifiable |  | 0.012 | 2.1 | 7.13 | 3.33 |  |  |  | 5.74, 38600 |
| 4307 | Non-Identifiable |  | 0.030 | 1.4 | 13.83 | 10.20 |  |  |  | 5.82, 39420 |
| 2308 | Non-Identifiable |  | 0.045 | 4.3 | 13.55 | 57.70 |  |  |  | 5.09, 36840 |
| 3825 | Non-Identifiable |  | 0.013 | 2.8 | 3.90 | 10.84 |  |  |  | 5.46, 185790 |
| 2107 | Non-Identifiable |  | 0.026 | 1.6 | 64.96 | 41.81 |  |  |  | 5.22, 24850 |
| 4724 | Non-Identifiable |  | 0.035 | 2.0 | 1.05 | 2.13 |  |  |  | 5.82, 88310 |
| 6309 | Non-Identifiable |  | 0.042 | 2.0 | 61.22 | 120.44 |  |  |  | 6.93, 39500 |

**Table S2**.

| **PD Quest spot number** | **UNIPROT accession number** | **Protein identification** | **p-value** | **Fold change** | **Average normalised quantity** | | **MASCOT score** | **Peptides matched** | **Sequence coverage (%)** | **pI, Mr (kDa)** |
| --- | --- | --- | --- | --- | --- | --- | --- | --- | --- | --- |
|  |  |  |  |  | **LFD** | **HFD** |  |  |  |  |
| 1213 | P63038 | *^ref18^ 60 kDa heat shock protein, mitochondrial | 0.000 | 4.2 | 3.86 | 16.18 | 47 | 8 (2) | 10 | 5.13, 32600 |
| 6704 | Q99KI0 | *^ref 3^ Aconitate hydratase, mitochondrial | 0.049 | 1.3 | 142.79 | 110.90 | 206 | 19 (10) | 25 | 7.94, 91650 |
| 3407 | P60710 | Actin, cytoplasmic 1 | 0.039 | 1.6 | 39.96 | 25.24 | 66 | 5 (3) | 13 | 6.03, 44270 |
| 3516 | Q8R1C9 | *^ref 6^Amyloid beta A4 precursor protein-binding family B member 3 | 0.036 | 1.8 | 24.51 | 13.76 | 58 | 5 (3) | 2 | 6.10, 60690 |
| 5510 | Q8R1C9 |  | 0.030 | 1.9 | 15.23 | 29.42 | 50 | 9 (1) | 5 | 7.08, 59040 |
| 3609 | Q8VBY2 | Calcium/calmodulin-dependent protein kinase kinase 1 | 0.006 | 3.2 | 35.47 | 10.99 | 76 | 3 (2) | 6 | 6.02, 68930 |
| 6316 | Q91V12 | Cytosolic acyl coenzyme A thioester hydrolase  Cytosolic acyl coenzyme A thioester hydrolase | 0.026 | 1.9 | 18.64 | 9.56 | 76 | 2 (2) | 6 | 7.52, 37160 |
| 5313 | Q91V12 |  | 0.049 | 1.6 | 23.99 | 14.97 | 161 | 7 (6) | 14 | 7.26, 37230 |
| 4511 | Q61753 | D-3-phosphoglycerate dehydrogenase | 0.049 | 2.2 | 12.30 | 26.58 | 135 | 4 (4) | 8 | 6.51, 60620 |
| 6202 | Q8BVI4 | Dihydropteridine reductase | 0.048 | 2.1 | 27.57 | 13.14 | 141 | 9 (6) | 33 | 7.67, 30630 |
| 3510 | O08553 | *^ref 10, 11^ Dihydropyrimidinase-related protein 2 | 0.031 | 1.5 | 580.88 | 388.03 | 346 | 36 (20) | 32 | 5.95, 62640 |
| 4606 | O08553 |  | 0.008 | 2.0 | 4.95 | 9.75 | 51 | 6 (2) | 15 | 6.24, 67130 |
| 3715 | P39053 | Dynamin-1 | 0.004 | 2.2 | 18.30 | 8.20 | 210 | 10 (8) | 12 | 6.09, 92930 |
| 4617 | Q9QXY6 | EH domain-containing protein 3 | 0.016 | 1.9 | 20.84 | 39.54 | 125 | 10 (6) | 19 | 6.52, 64520 |
| 2003 | Q9CQI3 | Glia maturation factor beta | 0.022 | 1.6 | 26.26 | 42.62 | 54 | 3 (1) | 26 | 5.23, 18170 |
| 5101 | P48774 | *^ref 12^Glutathione S-transferase Mu 5 | 0.001 | 1.3 | 34.78 | 46.18 | 128 | 10 (6) | 27 | 6.82, 27020 |
| 3207 | Q3UGR5 | Haloacid dehalogenase-like hydrolase domain-containing protein 2 | 0.036 | 1.3 | 26.81 | 20.07 | 41 | 2 (1) | 5 | 6.07, 30790 |
| 2715 | P07901 | Heat shock protein HSP 90-alpha | 0.025 | 2.8 | 36.59 | 103.25 | 93 | 7 (3) | 9 | 5.69, 95160 |
| 2701 | P11499 | Heat shock protein HSP 90-beta | 0.029 | 5.0 | 156.80 | 31.16 | 238 | 18 (10) | 22 | 5.20, 95120 |
| 5209 | Q99KB8 | Hydroxyacylglutathione hydrolase, mitochondrial | 0.006 | 2.4 | 11.28 | 26.79 | 52 | 4 (1) | 11 | 7.17, 29390 |
| 2102 | Q61171 | Peroxiredoxin-2 | 0.025 | 1.5 | 86.38 | 131.40 | 141 | 20 (8) | 38 | 5.20, 21940 |
| 2104 | P70296 | Phosphatidylethanolamine-binding protein 1 | 0.013 | 1.7 | 161.96 | 267.79 | 83 | 3 (3) | 18 | 5.26, 23000 |
| 5602 | Q9D0F9 | *^refs19, 20^ Phosphoglucomutase-1 | 0.026 | 1.9 | 24.71 | 12.68 | 111 | 12 (4) | 26 | 6.77, 63660 |
| 3409 | Q8BG07 | Phospholipase D4 | 0.011 | 2.5 | 13.17 | 5.33 | 40 | 2 (2) | 1 | 6.10, 47960 |
| 3206 | P23492 | Purine GTP-binding phosphorylase | 0.001 | 2.4 | 17.69 | 42.60 | 147 | 11 (6) | 37 | 6.06, 29810 |
| 2106 | Q99PT1 | Rho GDP-dissociation inhibitor 1 | 0.009 | 1.3 | 75.32 | 100.76 | 117 | 8 (5) | 26 | 5.30, 26080 |
| 2008 | P54227 | *^refs 21,22^Stathmin | 0.014 | 1.7 | 12.75 | 21.82 | 56 | 1 (1) | 8 | 5.69, 18320 |
| 5104 | P17751 | *^refs, 23, 24^Triose-phosphate isomerase | 0.009 | 1.6 | 37.04 | 60.04 | 125 | 9 (5) | 27 | 7.33, 27160 |
| 1206 | P21107 | Tropomyosin alpha-3 chain | 0.045 | 1.7 | 68.47 | 114.22 | 63 | 6 (3) | 15 | 4.88, 32270 |
| 2510 | Q7TMM9 | Tubulin beta-2A chain | 0.028 | 4.3 | 87.16 | 377.94 | 105 | 9 (7) | 22 | 5.69, 58720 |
| 1506 | P68372 | Tubulin beta-4B chain | 0.029 | 1.6 | 1173.09 | 1932.43 | 651 | 63 (35) | 48 | 5.07, 57200 |
| 3408 | Non-Identifiable |  | 0.008 | 4.1 | 16.21 | 3.99 |  |  | 1 | 6.09, 51950 |
| 3515 | Non-Identifiable |  | 0.049 | 2.0 | 14.23 | 7.16 |  |  |  | 6.09, 55570 |
| 1212 | Non-Identifiable |  | 0.028 | 3.3 | 10.34 | 33.82 |  |  |  | 5.01, 31930 |
| 3104 | Non-Identifiable |  | 0.033 | 1.3 | 12.59 | 15.91 |  |  |  | 5.97, 27630 |
| 4207 | Non-Identifiable |  | 0.046 | 2.0 | 7.30 | 14.84 |  |  |  | 6.61, 28850 |

**Table S3**

| **PD Quest spot number** | **UNIPROT accession number** | **Protein identification** | **p-value** | **Fold change** | **Average normalised quantity** | | **MASCOT score** | **Peptides matched** | **Sequence coverage (%)** |  |
| --- | --- | --- | --- | --- | --- | --- | --- | --- | --- | --- |
|  |  |  |  |  | **LFD** | **HFD** |  |  |  |  |
| 206 | P62259 | 14-3-3 protein epsilon | 0.038 | 1.7 | 419.02 | 253.31 | 685 | 76 (39) | 56 | 4.63, 29330 |
| 207 | P62259 |  | 0.022 | 2.0 | 92.08 | 181.39 | 607 | 47 (22) | 49 | 4.69, 29130 |
| 4502 | Q99JY9 | *^ref 4^Actin-related protein 3 | 0.008 | 1.4 | 40.10 | 56.40 | 91 | 11 (4) | 29 | 5.67, 50380 |
| 6501 | P17182 | *^refs 5^ Alpha-enolase | 0.029 | 1.4 | 86.65 | 63.91 | 158 | 11 (9) | 27 | 6.37, 47450 |
| 8409 | P05202 | Aspartate aminotransferase, mitochondrial | 0.048 | 1.8 | 90.18 | 160.67 | 186 | 18 (11) | 28 | 9.35, 40840 |
| 105 | Q91ZZ3 | *^ref 25,26^ Beta-synuclein | 0.022 | 1.2 | 63.89 | 75.94 | 74 | 3 (2) | 17 | 4.46, 19880 |
| 8 | P62204 | Calmodulin | 0.044 | 1.6 | 382.45 | 237.99 | 148 | 13 (8) | 60 | 4.09, 16870 |
| 5605 | P97427 | *^ref 9^ Dihydropyrimidinase-related protein 1 | 0.047 | 2.5 | 6.72 | 16.96 | 89 | 11 (3) | 15 | 6.22, 59520 |
| 3604 | O08553 | *^ref 10,11^ Dihydropyrimidinase-related protein 2 | 0.013 | 1.7 | 143.85 | 251.53 | 300 | 27 (12) | 35 | 5.54, 60430 |
| 6703 | Q80WG7 | E3 ubiquitin-protein ligase Trim36 | 0.015 | 1.5 | 15.72 | 10.47 | 48 | 3 (1) | 5 | 6.35, 75850 |
| 5505 | Q61553 | *^Refs17,27^Fascin | 0.028 | 1.4 | 57.54 | 81.12 | 89 | 6 (3) | 10 | 6.19, 51880 |
| 8306 | P05064 | Fructose-bisphosphate aldolase A | 0.040 | 1.9 | 800.62 | 430.71 | 216 | 20 (10) | 37 | 8.31, 39790 |
| 8501 | P97807 | Fumarate hydratase, mitochondrial | 0.033 | 2.2 | 75.60 | 34.92 | 80 | 9 (4) | 18 | 7.91, 45440 |
| 8205 | P10649 | *^Ref12^Glutathione S-transferase Mu 1 | 0.048 | 1.3 | 74.02 | 95.28 | 191 | 23 (11) | 66 | 8.27, 26260 |
| 6212 | O54879 | High mobility group protein B3 | 0.019 | 1.4 | 13.14 | 18.78 | 47 | 4 (1) | 10 | 6.72, 28310 |
| 1109 | P70296 | Phosphatidylethanolamine-binding protein 1 | 0.007 | 1.4 | 90.09 | 65.04 | 90 | 4 (3) | 26 | 4.98, 23070 |
| 17 | P50114 | Protein S100-B | 0.017 | 3.4 | 143.54 | 41.69 | 67 | 2 (1) | 16 | 4.47, 10050 |
| 4201 | P23492 | *^Refs 28,29^Purine nucleoside phosphorylase | 0.008 | 1.6 | 32.73 | 20.29 | 67 | 3 (2) | 7 | 5.74, 28840 |
| 3208 | P60487 | Pyridoxal phosphate phosphatase | 0.009 | 1.7 | 22.64 | 39.30 | 112 | 8 (4) | 32 | 5.56, 29480 |
| 2206 | P60487 |  | 0.045 | 1.9 | 30.87 | 57.63 | 209 | 11 (5) | 41 | 5.34, 29450 |
| 7606 | P52480 | *^Ref 30^ Pyruvate kinase PKM | 0.028 | 1.5 | 34.25 | 52.21 | 83 | 12 (4) | 18 | 7.50, 59210 |
| 5301 | Q93092 | Transaldolase | 0.017 | 1.3 | 26.98 | 20.99 | 116 | 10 (6) | 30 | 6.08, 36600 |
| 21 |  | Tubulin alpha-1A chain | 0.036 | 3.5 | 181.28 | 51.68 | 104 | 5 (2) | 5 | 4.59, 11430 |
| 1011 |  |  | 0.041 | 5.1 | 141.16 | 27.61 | 42 | 1 (1) | 1 | 4.88, 11610 |
| 2204 | Q9R0P9 | Ubiquitin carboxyl-terminal hydrolase isozyme L1 | 0.031 | 1.4 | 167.10 | 117.74 | 107 | 11 (5) | 42 | 5.25, 26550 |
| 6003 | P62983 | Ubiquitin-40S ribosomal protein S27a | 0.047 | 3.8 | 165.47 | 43.98 | 50 | 1 (1) | 5 | 6.60, 9780 |
| 1010 | Non-Identifiable |  | 0.034 | 2.0 | 46.43 | 23.74 |  |  |  | 4.88, 14710 |

**Table S4**

| **PD Quest spot number** | **UNIPROT accession number** | **Protein identification** | **p-value** | **Fold change** | **Average normalised quantity** | | **MASCOT score** | **Peptides matched** | **Sequence coverage (%)** | **pI, Mr (kDa)** |
| --- | --- | --- | --- | --- | --- | --- | --- | --- | --- | --- |
|  |  |  |  |  | **LFD** | **HFD** |  |  |  |  |
| 3305 | O88544 | COP9 signalosome complex subunit 4 | 0.005 | 0.76 | 47.90 | 36.56 | 214 | 20 (9) | 31 | 5.57, 46540 |
| 6707 | O08553 | *^refs 10,11^ Dihydropyrimidinase-related protein 2 | 0.010 | 1.49 | 11.52 | 17.11 | 44 | 1 (1) | 1 | 6.67, 74510 |
| 8324 | P05064 | Fructose-bisphosphate aldolase A | 0.014 | 0.54 | 256.88 | 137.66 | 517 | 36 (18) | 43 | 8.62, 46620 |
| 8309 | P05064 |  | 0.025 | 0.51 | 414.85 | 211.57 | 556 | 49 (23) | 75 | 8.31, 39790 |
| 6304 | P05063 | ^*Refs 31,32^Fructose-bisphosphate aldolase C | 0.005 | 1.82 | 18.88 | 34.37 | 133 | 8 (6) | 20 | 6.65, 41290 |
| 2904 | Q61699 | Heat shock protein 105 kDa | 0.008 | 0.6 | 115.88 | 70.01 | 456 | 29 (18) | 29 | 5.42, 120860 |
| 2804 | P11499 | Heat shock protein HSP 90-beta | 0.013 | 0.45 | 147.10 | 66.90 | 523 | 34 (23) | 31 | 5.42, 96540 |
| 2305 | O54983 | Ketimine reductase mu-crystallin | 0.003 | 2.21 | 83.00 | 183.56 | 857 | 38 (30) | 33 | 5.48, 37280 |
| 1104 | Q9CPU0 | *^ref 17^ Lactoylglutathione lyase | 0.022 | 1.62 | 55.97 | 90.77 | 216 | 32 (12) | 35 | 5.26, 23970 |
| 1705 | P08551 | Neurofilament light polypeptide | 0.019 | 1.38 | 29.44 | 40.70 | 209 | 26 (10) | 28 | 5.11, 77930 |
| 8424 | Q9CZ30 | Obg-like ATPase 1 | 0.018 | 0.3 | 181.09 | 55.02 | 69 | 12 (5) | 15 | 8.60, 48080 |
| 1122 | Q61171 | Peroxiredoxin-2 | 0.006 | 1.8 | 105.94 | 191.21 | 400 | 25 (18) | 35 | 5.20, 21940 |
| 119 | Q61171 |  | 0.020 | 1.59 | 49.26 | 78.50 | 194 | 16 (10) | 26 | 5.08, 22220 |
| 5720 | Non-Identifiable |  | 0.010 | 1.59 | 13.27 | 21.06 |  |  |  | 6.34, 86480 |

**Table S5**

| **Reactome pathway identifier** | **Pathway name** | **Number of proteins detected in pathway** | **Total number of proteins in pathway** | **Mapped proteins** |
| --- | --- | --- | --- | --- |
| R-MMU-1430728 | Metabolism | 12 | 1955 | P19157; Q99KI0; Q9CPU0; Q9D6R2; Q8BG07; Q9D1A2; P17182; P62204; Q04447; P17183; P05064; P09411 |
| R-MMU-162582 | Signal transduction | 8 | 2771 | P68369; P50396; Q76MZ3; P05213; Q3UHC7; P62204; Q61224; P63085 |
| R-MMU-422475 | Axon guidance | 6 | 516 | Q76MZ3; P97427; O08553; Q3UHC7; P62204; P63085 |
| R-MMU-1266738 | Developmental biology | 6 | 740 | Q76MZ3; P97427; O08553; Q3UHC7; P62204; P63085 |
| R-MMU-70326 | Glucose metabolism | 5 | 95 | P17182; P62204; P17183; P09411; P05064 |
| R-MMU-76002 | Platelet activation, signalling and aggregation | 5 | 271 | P20029; Q921I1; P62204; P63085; P05064 |
| R-MMU-71387 | Metabolism of carbohydrates | 5 | 317 | P17182;P62204;P17183;P09411;P05064 |
| R-MMU-109582 | Haemostasis | 5 | 592 | P20029;Q921I1;P62204;P63085;P05064 |
| R-MMU-1640170 | Cell cycle | 5 | 646 | P68369;O35144;Q76MZ3;P63085;P17156 |
| R-MMU-372790 | Signalling by GPCR | 5 | 1395 | Q76MZ3;Q3UHC7;P62204;Q61224;P63085 |
| R-MMU-168256 | Immune system | 5 | 1399 | P26011;Q76MZ3;Q3UHC7;P62204;P63085 |

**Table S6**

| **Reactome pathway identifier** | **Pathway name** | **Number of proteins detected in pathway** | **Total number of proteins in pathway** | **Mapped proteins** |
| --- | --- | --- | --- | --- |
| R-MMU-1430728 | Metabolism | 8 | 1955 | Q99KI0; Q61753; P07901; Q9D0F9; Q8BG07; Q91V12; P17751; Q8BVI4 |
| R-MMU-422475 | Axon guidance | 6 | 516 | P70296; P07901; P60710; O08553; P11499; P39053 |
| R-MMU-1266738 | Developmental Biology | 6 | 740 | P70296; P07901; P60710; O08553; P11499; P39053 |
| R-MMU-162582 | Signal Transduction | 6 | 2771 | P70296; P07901; P60710; Q99PT1; Q7TMM9; P68372 |
| R-MMU-168249 | Innate Immune System | 5 | 780 | P70296; P07901; P60710; P11499; P39053 |
| R-MMU-168256 | Immune System | 5 | 1399 | P70296; P07901; P60710; P11499; P39053 |

**Table S7**

| **Reactome pathway identifier** | **Pathway name** | **Number of proteins detected in pathway** | **Total number of proteins in pathway** | **Mapped proteins** |
| --- | --- | --- | --- | --- |
| R-MMU-1430728 | Metabolism | 6 | 1955 | Q93092;P52480;P05202;P62204;P97807;P05064 |
| R-MMU-162582 | Signal Transduction | 6 | 2771 | P68369;P70296;P50114;P62259;Q99JY9;P62204 |
| R-MMU-71387 | Metabolism of carbohydrates | 5 | 317 | Q93092;P52480;P05202;P62204;P05064 |
| R-MMU-422475 | Axon guidance | 5 | 516 | P70296;P97427;Q99JY9;O08553;P62204 |
| R-MMU-1266738 | Developmental Biology | 5 | 740 | P70296;P97427;Q99JY9;O08553;P62204 |
| R-MMU-168256 | Immune System | 5 | 1399 | P70296;Q80WG7;P50114;Q99JY9;P62204 |

| **Reactome pathway identifier** | **Pathway name** | **Number of proteins detected in pathway** | **Total number of proteins in pathway** | **Mapped proteins** |
| --- | --- | --- | --- | --- |
| R-MMU-1430728 | Metabolism | 4 | 1955 | Q9CPU0;O54983;P05064;P05063 |
| R-MMU-2262752 | Cellular responses to stress | 3 | 326 | Q61171;P11499;Q61699 |

**Table S8**
