# Supplementary figures and images for: Early and reversible changes to the hippocampal proteome in mice on a high-fat diet
Source: Nutr Metab (Lond). 2019 Aug 23;16:57. doi: 10.1186/s12986-019-0387-y (PMC6708244; doi:10.1186/s12986-019-0387-y)

Supplementary Figure 1

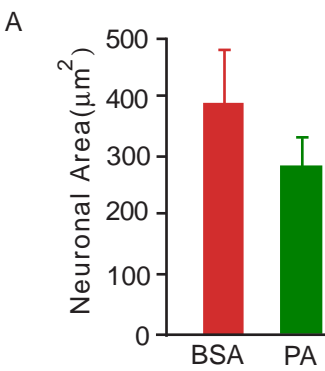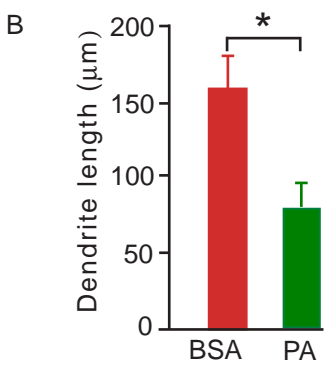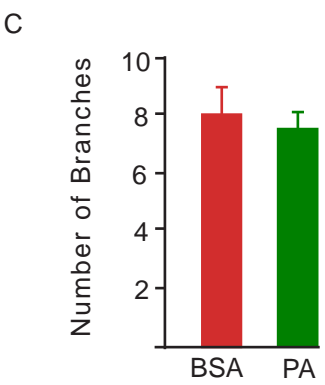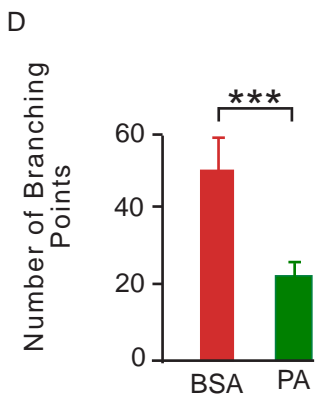

Supplement: Supplementary file 2 — Figure S1. A. Area of MAP2 immunostaining in primary hippocampal neurons in culture B. Dendrite length C. Mean number of dendritic branches originating from soma D. Number of dendritic branching points. Neurons were challenged with BSA (control) or 200 μm palmitic acid PA solutions were changed and cells washed followed by a subsequent 3 h treatment with the same challenge (* p < 0.05, *** p < 0.001). Data presented as mean ± SEM, n = 10 all groups. Bovine serum albumin (BSA), palmitic acid (PA) and docosahexaenoic acid (DHA), microtubule-associated protein 2 (MAP2). (PDF 9 kb) [file 12986_2019_387_MOESM2_ESM.pdf]

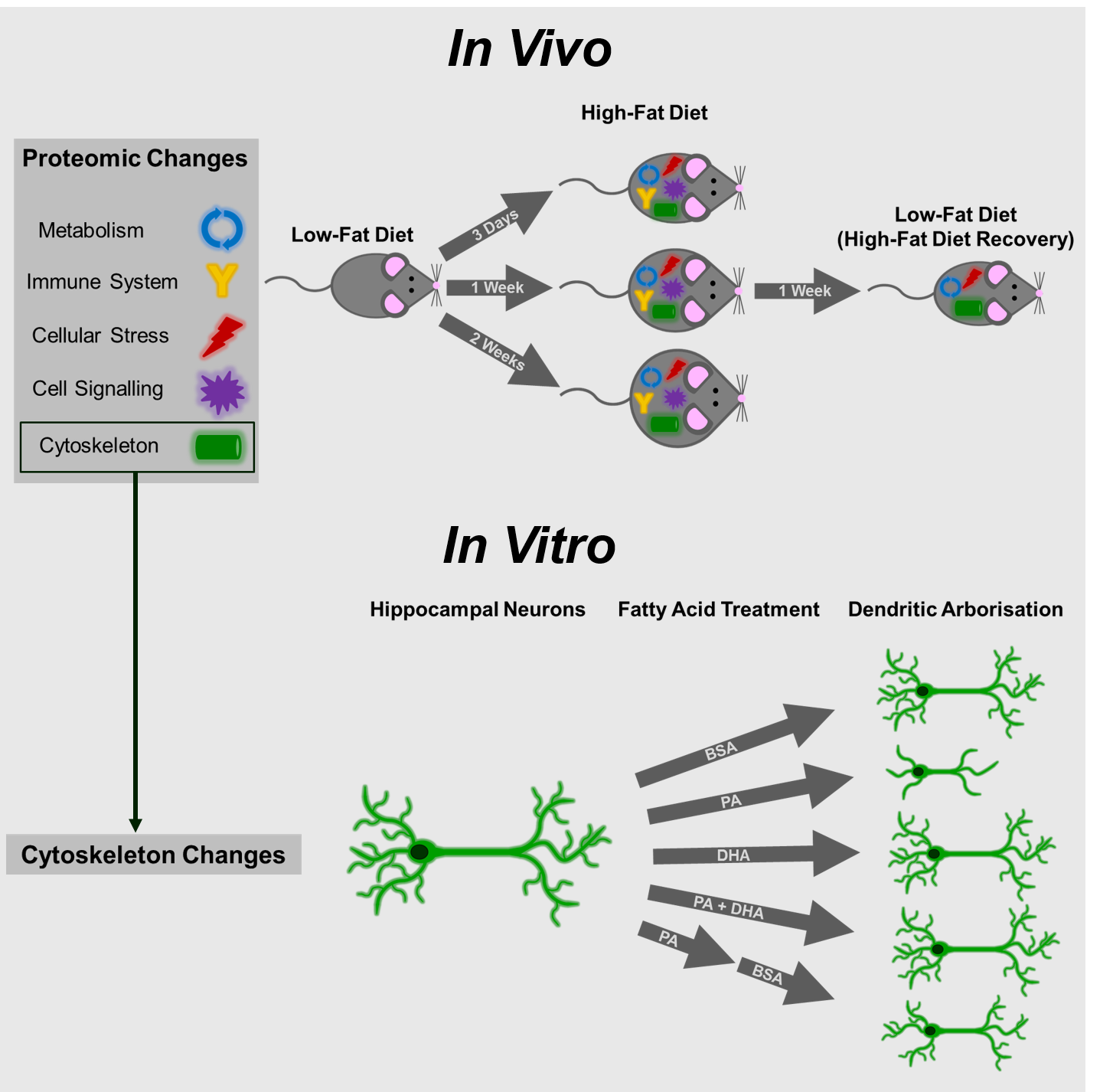

Supplement: Supplementary file 3 — Figure S2. A diagrammatic representation of the experimental design and major outcomes of the study. (TIF 624 kb) [file 12986_2019_387_MOESM3_ESM.tif]
