# Supplementary material for: Early and reversible changes to the hippocampal proteome in mice on a high-fat diet
Source: Nutr Metab (Lond). 2019 Aug 23;16:57. doi: 10.1186/s12986-019-0387-y (PMC6708244; doi:10.1186/s12986-019-0387-y)
Supplement: Supplementary file 4 — References for Tables S1-S4. (DOCX 23 kb) [file 12986_2019_387_MOESM4_ESM.docx]

**Supplementary References**

**1.** [Garcia-Alloza](https://www.nature.com/articles/1300330#auth-1), M., [Hirst](https://www.nature.com/articles/1300330#auth-2) W D., [Chen](https://www.nature.com/articles/1300330#auth-3) C P L-H, [Lasheras](https://www.nature.com/articles/1300330#auth-4), B, [Francis](https://www.nature.com/articles/1300330#auth-5) P T., [Ramírez](https://www.nature.com/articles/1300330#auth-6) M J Differential Involvement of 5-HT_1B/1D_ and 5-HT_6_ Receptors in Cognitive and Non-cognitive Symptoms in Alzheimer's Disease *Neuropsychopharmacology* 29, 410–416(2004)

**2.** [Yoo,](https://www.sciencedirect.com/science/article/pii/S0006291X00941093#!) C.B.[,](https://www.sciencedirect.com/science/article/pii/S0006291X00941093#!) Kim S.H., [Cairns^,^](https://www.sciencedirect.com/science/article/pii/S0006291X00941093#!) N., [Fountoulakis^,^](https://www.sciencedirect.com/science/article/pii/S0006291X00941093#!) M.,[Lubec](https://www.sciencedirect.com/science/article/pii/S0006291X00941093#!), G.,Deranged Expression of Molecular Chaperones in Brains of Patients with Alzheimer's Disease. [Biochemical and Biophysical Research Communications](https://www.sciencedirect.com/science/journal/0006291X), 2001;280: 249-258

**3.** Shen L, Chen C, Yang A, Chen Y, Liu Q, Ni J. Redox proteomics identification of specifically carbonylated proteins in the hippocampi of triple transgenic Alzheimer's disease mice at its earliest pathological stage. Journal of proteomics. 2015;123:101-13.

**4**.Ottis P, Loos M, Li KW, de Souza A, Schulz D, Smit AB, et al. Aging-Induced Proteostatic Changes in the Rat Hippocampus Identify ARP3, NEB2 and BRAG2 as a Molecular Circuitry for Cognitive Impairment. PloS one. 2013;8:e75112.

**5.** Butterfield DA, Lange MLB. Multifunctional roles of enolase in Alzheimer’s disease brain: beyond altered glucose metabolism. J Neurochem. 2009;111:915-33.

**6.** Csiszar A, Tucsek Z, Toth P, Sosnowska D, Gautam T, Koller A, et al. Synergistic effects of hypertension and aging on cognitive function and hippocampal expression of genes involved in beta-amyloid generation and Alzheimer's disease. Am J Physiol Heart Circ Physiol. 2013;305:H1120-30.

**7.** David S., Shoemaker M., Haley B. E. Abnormal properties of creatine kinase in Alzheimer's disease brain: correlation of reduced enzyme activity and active site photolabeling with aberrant cytosol-membrane partitioning. *Molecular Brain Research*. 54, 276-87 (1998).

**8.** Castegna A., Aksenov M., Aksenova M., Thongboonkerd V., Klein J. B., Pierce W. M., Booze R., Markesbery W. R., Butterfield D. A. Proteomic identification of oxidatively modified proteins in Alzheimer’s disease brain. Part I: creatine kinase BB, glutamine synthase, and ubiquitin carboxy-terminal hydrolase L-1. *Free Radical Biology and Medicine*. 33, 562-71 (2002).

**9.** Chang RYK, Etheridge N, Dodd PR, Nouwens AS. Targeted quantitative analysis of synaptic proteins in Alzheimer’s disease brain. Neurochem Int. 2014;75:66-75.

**10.** Castegna A, Aksenov M, Thongboonkerd V, Klein JB, Pierce WM, Booze R, et al. Proteomic identification of oxidatively modified proteins in Alzheimer's disease brain. Part II: dihydropyrimidinase‐related protein 2, α‐enolase and heat shock cognate 71. J Neurochem. 2002;82:1524-32.

**11.** Cole A. R., Noble W., Aalten L. v., Plattner F., Meimaridou R., Hogan D., Taylor M., LaFrancois J., Gunn‐Moore F., Verkhratsky A. Collapsin response mediator protein‐2 hyperphosphorylation is an early event in Alzheimer’s disease progression. *Journal of neurochemistry*. 103, 1132-44 (2007).

**12.** Lovell M., Xie C., Markesbery W. Decreased glutathione transferase activity in brain and ventricular fluid in Alzheimer's disease. *Neurology*. 51, 1562-6 (1998)

**13.** Fontaine SN, Martin MD, A Dickey CA, Neurodegeneration and the Heat Shock Protein 70 Machinery: Implications for Therapeutic Development. Current topics in medicinal chemistry. 2016;16:2741-52.

**14.** Repalli J, Meruelo D. Screening strategies to identify HSP70 modulators to treat Alzheimer's disease. Drug Des Devel Ther. 2015;9:321-31

**15.** Bubber P, Haroutunian V, Fisch G, Blass JP, Gibson GE. Mitochondrial abnormalities in Alzheimer brain: mechanistic implications. Ann Neurol. 2005;57:695-703.

**16.** Naseri NN, Xu H, Bonica J, Vonsattel JPG, Cortes EP, Park LC, et al. Abnormalities in the tricarboxylic Acid cycle in Huntington disease and in a Huntington disease mouse model. Journal of Neuropathology & Experimental Neurology. 2015;74:527-37.

**17.** Zahid S, Oellerich M, Asif AR, Ahmed N. Differential expression of proteins in brain regions of Alzheimer’s disease patients. Neurochem Res. 2014;39(1):208-15.

**18.** Marino Gammazza A, Caruso Bavisotto C, Barone R, Macario ECd, JL Macario A. Alzheimer’s disease and molecular chaperones: current knowledge and the future of chaperonotherapy. Curr Pharm Des. 2016;22:4040-9.

**19.** Minjarez B, Calderón-González KG, Rustarazo MLV, Herrera-Aguirre ME, Labra-Barrios ML, Rincon-Limas DE, et al. Identification of proteins that are differentially expressed in brains with Alzheimer's disease using iTRAQ labeling and tandem mass spectrometry. Journal of proteomics. 2016;139:103-21.

**20.** Ding B, Xi Y, Gao M, Li Z, Xu C, Fan S, et al. Gene expression profiles of entorhinal cortex in Alzheimer’s disease. American Journal of Alzheimer's Disease & Other Dementias®. 2014;29:526-32.

**21.** Shumyatsky GP, Malleret G, Shin R, Takizawa S, Tully K, Tsvetkov E, et al. Stathmin, a gene enriched in the amygdala, controls both learned and innate fear. Cell. 2005;123:697-709.

**22.** Chauvin S, Sobel A. Neuronal stathmins: a family of phosphoproteins cooperating for neuronal development, plasticity and regeneration. Prog Neurobiol. 2015;126:1-18.

**23.** Guix F. X., Ill-Raga G., Bravo R., Nakaya T., de Fabritiis G., Coma M., Miscione G. P., Villà-Freixa J., Suzuki T., Fernandez-Busquets X. Amyloid-dependent triosephosphate isomerase nitrotyrosination induces glycation and tau fibrillation. *Brain*. 132, 1335-45 (2009).

**24.** Tajes M., Eraso-Pichot A., Rubio-Moscardó F., Guivernau B., Ramos-Fernández E., Bosch-Morató M., Guix F., Clarimón J., Miscione G., Boada M. Methylglyoxal Produced by Amyloid-Peptide-Induced Nitrotyrosination of Triosephosphate Isomerase Triggers Neuronal Death in Alzheimer’s Disease. *Journal of Alzheime*

**25.** Oeckl,P., Metzger,F., Nagl,M., von Arnim,C.A.F , Halbgebauer,S., Steinacker,P., Ludolph,A.C., Otto,M. Alpha-, beta- and gamma-synuclein quantification in cerebrospinal fluid by multiple reaction monitoring reveals increased concentrations in Alzheimer′s and Creutzfeldt-Jakob disease but no alteration in synucleinopathies. Molecular & Cellular Proteomics 2016, mcp.M116.059915; <https://doi.org/10.1074/mcp.M116.059915>

**26.** Castaño EM, Maarouf CL, Wu T, Leal MC, Whiteside CM, Lue L, et al. Alzheimer disease periventricular white matter lesions exhibit specific proteomic profile alterations. Neurochem Int. 2013;62:145-56

**27.** Wei Z, Sun M, Liu X, Zhang J, Jin Y. Rufy3, a protein specifically expressed in neurons, interacts with actin‐bundling protein Fascin to control the growth of axons. J Neurochem. 2014;130:678-92.

**28.** Peña-Altamira LE, Polazzi E, Giuliani P, Beraudi A, Massenzio F, Mengoni I, et al. Release of soluble and vesicular purine nucleoside phosphorylase from rat astrocytes and microglia induced by pro-inflammatory stimulation with extracellular ATP via P2X7 receptors. Neurochem Int. 2017.115:37-49

**29.** Garcia‐Esparcia P, Hernández‐Ortega K, Ansoleaga B, Carmona M, Ferrer I. Purine metabolism gene deregulation in Parkinson's disease. Neuropathol Appl Neurobiol. 2015;41:926-40

**30.** Harris RA, Tindale L, Cumming RC. Age-dependent metabolic dysregulation in cancer and Alzheimer’s disease. Biogerontology. 2014;15:559-77.

**31.** Halford J, Shen S, Itamura K, Levine J, Chong AC, Czerwieniec G, et al. New astroglial injury-defined biomarkers for neurotrauma assessment. Journal of Cerebral Blood Flow & Metabolism. 2017;37:3278-99.

**32.** Oppelt SA, Zhang W, Tolan DR. Specific regions of the brain are capable of fructose metabolism. Brain Res. 2017;1657:312-22.
